# Parental survey of the sleep patterns and screen time in US school children during the first 6 months of the COVID-19 pandemic

**DOI:** 10.1186/s12887-023-03875-9

**Published:** 2023-02-08

**Authors:** Amanda B. Hassinger, Alberto Monegro, Geovanny Perez

**Affiliations:** 1 Department of Pediatrics, Division of Pulmonary and Sleep Medicine, University at Buffalo Jacobs School of Medicine and Biomedical Sciences, 1001 Main Street, 5Th Floor, Buffalo, NY 14203 USA; 2 Department of Internal Medicine and Pediatrics, Division of Pulmonary, Critical Care and Sleep Medicine, University at Buffalo Jacobs School of Medicine and Biomedical Sciences, Buffalo, NY USA

**Keywords:** Sleep, Pandemic, Children, Sleep patterns, School closures, Screen time

## Abstract

**Background:**

This study compared sleep duration, screen exposure and sleep quality in school-aged children before COVID-19 to that during school closures and again when schools re-opened in fall 2020.

**Methods:**

Cross-sectional anonymous, online survey of parents of children 5–13 years old. Questions elicited information about sleep timing and quality, screen time, and schooling at three distinct periods: before the pandemic, when schools first closed and then re-opened in the fall.

**Results:**

Respondents described 101 children who were an average of 8.5 years old and 51% male. In lockdown, children slept 25 min more (95%CI 00:13–00:38) due to later wake times (75 min, 95% CI 0:57–1:34) with later bedtimes (29 min, 95%CI 0:00–0:58). When schools re-opened, sleep duration returned to pre-pandemic levels, but sleep onset and offset times remained later. Despite more sleep, sleep quality and habits (e.g. bedtime refusal) worsened during lockdown and did not normalize in fall 2020. During lockdown, screen time increased in 65% of all children, and 96% of those in private schools. When schools reopened, 78% of children in hybrid/virtual learning had more than 4 h of screen exposure daily. Less screen time was associated with twofold higher odds of better sleep (OR 2.66, 95%CI 1.15–6.14).

**Conclusions:**

Although school-aged children had more total sleep when schools were closed, sleep quality and habits worsened. Upon return to school, sleep times and quality did not normalize and were linked to screen time.

**Supplementary Information:**

The online version contains supplementary material available at 10.1186/s12887-023-03875-9.

## Background

At the onset of the COVID-19 (coronavirus disease 2019) pandemic, schools were closed abruptly and millions of school-aged children throughout the world were isolated at home [1]. As children were no longer anchored to a regular morning routine controlled by bus schedules or school start times, there was an immediate disruption in daily sleep schedules. Large international studies have shown the pandemic disrupted the sleep of 40% of the world’s population [2]. Early data from the first outbreak areas found this extended to children as well [3, 4]. In one study, half of all school-aged children were going to bed later and waking up later, leading to more sleep overall [5].

Few studies have investigated if more sleep during lockdown meant higher sleep quality for children at the time. One possible reason children’s sleep quality could have worsened was the marked increase in screen time. Pre-pandemic studies describing the recent trend towards increased screen use and social media exposure in youth and adolescents have found troubling connections to poor sleep quality and negative effects on school performance [6–9]. During the lockdown, educational models reverted to entirely online platforms and all in-person social interactions were cancelled, leading to markedly increased amounts of daily screen time for children [10]. One study reported the average increase in screen time in children was by 4 h per day during the pandemic [3]; an amount which is itself double the recommended daily screen time by the American Academy of Pediatrics [11]. While studies have tied this increase in screen time in COVID-19 to mental health [12–14], its impact on the sleep habits of children during this ongoing pandemic is not well described.

Annually, the change in sleep schedules when school restarts every fall is an expected disruption to daily life. This is best illustrated in the study of children when they begin high school with earlier school times in the setting of physiologically later bed times leading to insufficient sleep, poor school performance and significant impact on quality of life [15, 16]. When schools restarted on the fall of 2020, the landscape of school schedules and learning models were even more challenging than usual because of the nature of the ongoing pandemic. Depending on physical class sizes, local mandates and school district resources, school formats ranged from entirely virtual to hybrid to all in-person. For the majority of school-aged children, there were still no daily in-person class start times dictating morning commute, so irregular wake times from the spring school closures likely continued. While little data are available describing the sleep patterns of children after a 6-month lockdown, it is imperative to determine how to assist school-aged children in maintaining healthy sleep habits during this ongoing health crisis and during future disruptions in school schedules that could derail day-to-day routines. Further, investigating how the pandemic has impacted the sleep health of children has been identified as an important area of focus to mitigate long-term damage to childhood development and quality of life [17–19]. Even small shifts in the established daily routines can detrimentally affect the normal circadian rhythm leading to “social jet lag” and affecting overall health and wellness [20]. Irregular sleep schedules, duration and quality could be part of the reason why more adolescents and young adults have been suffering from mental health disorders, including insomnia, during this pandemic [21–23].

This study was performed to test the hypothesis that the school closures in the beginning of the pandemic would be associated with a later bed time, later wake time and longer overall sleep *duration* in school-aged children but not improved sleep *quality*. We further hypothesized that the sleep patterns would not revert back to normal when the school year began in the fall of 2020 because of altered educational formats. This study included children attending public, private or home-school to allow for consideration of any socio-economic and scheduling confounders that could be related to sleep patterns. The intention was to identify children whose sleep was most impacted as important targets for education and potential intervention in this and future public health crises.

## Methods

### Study design and dissemination

This was a single, anonymous, cross-sectional survey accessible via an electronic link or QR code to the affiliated university’s RedCap website (Research Electronic Data Capture, LLC). The Institutional Review Board of the University at Buffalo Jacobs School of Medicine and Biomedical Sciences approved the project with a modified consent process to protect subject anonymity.

Eligible participants were parents or guardians of school-aged children in the local area. This included any child age 5 to 13 years old when the survey was administered in December 2020.

The intended distribution was by individual school districts through student body email lists; this did occur in three private schools. However, another surge of the virus overwhelmed local schools at the same time as study roll-out, limiting the pragmatism of this method. Posted QR codes in local pediatric clinics, word-of-mouth and public health social media posts were used to recruit instead. To reach home-schooled children, a link to the study with a brief description was posted on a frequently used home-schooling website, Classical Conversations (https://www.classicalconversations.com). This recruitment strategy was intended to capture a convenience sample of school children in our region.

### Pandemic timeline related to the survey in the local area

In New York, one of the originally hard hit states in the United States, the local schools were closed on March 13, 2020 and did not open again for any in-person learning, even hybrid, until September 2020. For approximately 10 weeks, all non-essential business were also closed, including restaurants, places of worship and daycare centers, in accordance with the state mandates until the first wave subsided. This is the period referred to as “lockdown” in the survey.

Schools did re-open partially for the fall semester in 2020. This study was distributed 3 months after this school year began, December 2020. In the local area, this coincided with a second COVID infection peak. At the time the survey was disseminated, new COVID cases were numbering 100 s per day with 10 to 20 deaths daily and local hospitals were near or at full capacity.

### Survey content

The introduction screen was an explanation of informed consent to the respondents which was implied upon survey entrance. Demographics collected included child age, sex, home structure and annual family income. Using a single survey at one time, respondents described their child’s school type, screen time, bedtime and wake time on both weekends and weekdays before the pandemic, during lockdown and during the start of the school year in 2020. These are similar phases of the pandemic used to describe the mental health and quality of life in other student populations [24]. (Survey available as Supplement 1).

Parents were asked to report how often their children exhibited healthy sleep habits across six aspects of the Children Sleep Health Questionnaire [25]: “Went to bed at the same time,” “Slept in a parent’s or sibling’s bed,” “Had a bad dream or nightmare,” “Struggled at bedtime,” “Tired or hyperactive during the day,” or “Did not get enough sleep”. A three-point Likert scale (“Rarely”, “Sometimes” or “Usually”) was used to quantify frequency of these sleep patterns before the pandemic, during lockdown and then during the school year. Respondents ranked the quality of their child’s sleep from “Poor” to “Excellent” at each of the three phases of the pandemic.

### Statistical analysis

As an anonymous survey, answers were reviewed for plausibility but ensuring data reliability was not possible. Any surveys which did not contain more than one response were omitted. No questions were mandatory so missing answers were omitted. Data were summarized as proportions of participants responding to each question. Paired t-tests compared parametric data within participants; one-sided ANOVAs compared multiple variables. Chi-square or Fisher’s Exact tests compared categorical variables between groups; logistic regression analyses controlled for confounders. Analyses were performed using IBM SPSS software, version 26 (Chicago, IL) with significance set at a *p*-value < 0.05 using two-sided comparisons.

## Results

### Patient population

Of the 105 surveys started, 4 were empty and omitted. The majority of the respondents’ children were less than 9 years old (65%) and lived in a home with two parents (86.1%). Almost half were in private school, 35% in public and 12% were home-schooled before the pandemic (Table 1).

**Table 1:** Description of the children of survey respondents

Variable	Categories	Total population (*n* = 101)
Mean age (years)		8.5 (± 2.6)
Male sex n(%)		52 (53%)
Home family structure	Single parent	5 (5%)
	2 parent home	87 (86%)
	Split time between homes	7 (7%)
Income level	$25,000–99,999	26 (26%)
	$100–149,999	30 (30%)
	$150–249,999	22 (22%)
	> $250,000	15 (15%)
Parent is an essential worker		61 (60%)
Essential worker is in health care		26/51 (26% of total population)
School type before the pandemic	No school yet (< 5 years old)	5 (5%)
	Public	35 (35%)
	Private	47 (47%)
	Home schooled	12 (12%)

Data are presented as mean (± SD) or count (proportion of column or of respondents to each survey item)

### Total sleep time

Average total sleep time (TST) per night was 10.34 (± 0.79) hours before the pandemic, 10.76 (± 1.07) hours during lockdown and 10.36 (± 0.98) hours when the school year started in fall 2020. Average TST increased by 0.42 h or 25 min (95%CI 0.21–0.64, *p* < 0.001) from before the pandemic to during lockdown. Average TST then fell by 0.36 h or 22 min (95%CI 0.12–0.60, *p* = 0.003) from lockdown to the start of the school year. There was no significant difference between TST before the pandemic and during the school year (difference 0.04 h, 95%CI -0.11 to 0.19, *p* = 0.613).

There was a negative linear association between age and TST before the pandemic (*r* = -0.458, *p* < 0.001). This correlation lessened during lockdown, *r* = -0.168, *p* = 0.051 and returned when the school year started again (*r* = -0.377, *p* < 0.001). This trend suggests that older students slept more on lockdown but when school restarted, they woke up sooner, shortening the TST.

### Bedtime and wake times

The average bedtime before the pandemic was 20:31 (± 0:46) on weekdays and 21:11 (± 1:06) on weekends. This increased to 21:01 (± 2:22) on lockdown weekdays and 21:46 (± 1:36) on lockdown weekends. When school restarted in the fall 2020, the average bedtime was 20:52 (± 1:01) on weekdays and 21:26 (± 1:18) on weekends.

There was a statistically significant change in the average bedtime to 29 min later on weekdays (0:29, 95%CI 0:00–0:58, *p* = 0.046) and 34 min later on weekends (0:34, 95%CI 0:23–0:46, *p* < 0.001) when comparing before the pandemic to the lockdown period. When comparing the average bedtime during lockdown to when school started in the fall of 2020, weekday bedtime did not change significantly (-0:07, 95%CI -0:37 to 0:22, *p* = 0.636), but weekend bedtime did occur 18 min earlier (-0:18, 95%CI -0:34 to -0:02, *p* = 0.024) when school started. Overall, the average bedtime on weekdays was 21 min later when school restarted then compared to before the pandemic (0:21, 95%CI 0:14–0:28, *p* < 0.001) and 15 min later on weekends (0:15, 95%CI 0:03–0:27, *p* = 0.010).

The average wake time for all subjects before the pandemic was 6:45am (± 0:42) on weekdays and 7:47am (± 1:17) on weekends. This increased to 8:01am (± 1:32) on weekdays and 8:19am (± 1:44) on weekends during lockdown. When school started, the average wake time on weekdays was 7:06am (± 1:01) and on weekends was 8:06 (± 1:36). All comparisons of wake time from before the pandemic to during the lockdown to during the school year on weekends and weekdays was statistically significant (all *p* values 0.001 or less).

### Parental reports of sleep habits

The majority of respondents reported that before the pandemic their children were doing well with going to bed at the same time every night (87%), sleeping independently (82%), not struggling at bedtime (89%), not having nightmares (92%) and getting adequate sleep (68%). Every aspect of these subjective assessments of sleep habits worsened during lockdown and did not return to pre-pandemic levels during the fall of 2020 (Table 2).

**Table 2:** Parental report of school-aged children’s sleep quality and behaviors before COVID-19, during the school closures (lockdown) and during the school year in the fall of 2020

Sleep habit	**Before** the pandemic	**Lockdown**	**School year** in fall 2020	Comparing **before** to **lockdown**	Comparing **lockdown** to **fall 2020**	Comparing **fall 2020** to **before**
USUALLY or ALWAYS Went to bed the same time every night	87%	39%	58%	0.025^a^	< 0.001^a^	< 0.001^a^
RARELY Slept in another’s bed	82%	69%	72%	< 0.001^a^	< 0.001	< 0.001
RARELY Had a nightmare	92%	67%	75%	0.002^a^	< 0.001	0.003^a^
RARELY Struggled at bedtime	89%	65%	70%	< 0.001^a^	< 0.001^a^	0.014^a^
RARELY Tired or hyperactive during the day	65%	40%	43%	< 0.001^a^	< 0.001	< 0.001^a^
RARELY Did not get enough sleep	68%	46%	47%	< 0.001^a^	< 0.001	< 0.001^a^
Had GOOD or EXCELLENT Sleep	90%	48%	58%	0.060^a^	< 0.001	0.016^a^

Data are shown as the proportion of respondents who chose the frequency of each behavior described on the left most column. Chi-square testing used unless cell count mandated Fisher’s Exact Testing^a^

Before the pandemic, 9 in 10 parents reported that their child’s sleep was good or excellent. This dropped to 48% of parents during lockdown and increased to 58% during the start of the 2020 school year.

### Reported screen time

**Fig. 1:**
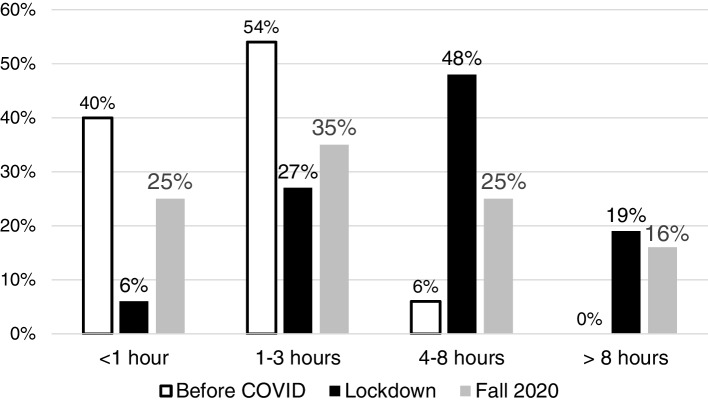
Proportion of children whose parents reported daily screen time in each amount listed here. The white bars represent the screen time before the pandemic, black during the spring of 2020 when schools were all closed (“lockdown”) and gray during the restart of schools in the fall of 2020

The proportion of parents who reported their child had more than 4 h of screen use per day went from 6% before the pandemic to 67% during lockdown and to 40% once school restarted in the fall of 2020. Screen use for more than 8 h a day occurred in 0% of children before the pandemic, in 19% during lockdown and 16% in the fall of 2020 (Fig. 1). The change from before the pandemic to during the school year was statistically significant (*p* = 0.003) as was the decrease from lockdown to when school started (*p* < 0.001). Only 13% of parents reported that screen use before the pandemic was related to education, this increased to 52% during lockdown and 43% during the school year.

### Comparing types of schools

The breakdown of demographics and sleep parameters for the children by school type before the pandemic and during lockdown are described in Table 3. Before the pandemic, homeschoolers slept longer (*p* = 0.057) and woke up later when compared to those in private, public or not in school yet (*p* = 0.006). Overall the bedtime and wake times varied less between weekends and weekdays in children who were home-schooled than those in public or private school before the pandemic. A pattern that persisted even during lockdown. The differences by school type in total sleep time and wake time did disappear once the schools were closed. Of note, the average bedtime during lockdown for those children in public school was significantly later (22:20 ± 1:44) on weekdays than that of children in private schools (21:44 ± 1:31) or home-schooled (20:50 ± 0:39), *p* = 0.010.

**Table 3:** Comparison of children as categorized by school-type before the COVID-19 pandemic began

Variable	Categories	Not in school yet (*n* = 5)	In public school (*n* = 35)	In private school (*n* = 47)	Home schooled (*n* = 12)	*p*-value
Mean age (years)		5.2 (± 0.45)	9.3 (± 2.9)	8.4 (± 2.5)	8 (± 1.8)	0.004
Male sex n(%)		3 (60%)	20 (57%)	20 (44%)	9 (75%)	0.226
Home family structure	Single parent	0%	3 (9%)	2 (4%)	0%	0.377
	2 parent home	5 (100%)	31 (89%)	39 (83%)	12 (100%)	
	Split time between homes	0%	1 (3%)	6 (13%)	0%	
Income level	$25,000–99,999	1 (20%)	9 (27%)	13 (28%)	3 (25%)	0.446
	$100–149,999	1 (20%)	11 (32%)	11 (23%)	7 (58%)	
	$150–249,999	2 (40%)	6 (18%)	14 (30%)	0%	
	> $250,000	1 (20%)	7 (21%)	7 (15%)	0%	
Parent is an essential worker		2 (40%)	25 (74%)	25 (56%)	9 (75%)	0.205
Essential worker is in health care		1 (20%)	12 (35%)	11 (24%)	2 (17%)	0.957
**SLEEPING PATTERNS BEFORE THE PANDEMIC**						
Bedtime on weekdays		19:54 (± 0:25)	20:42 (± 0:48)	20:29 (± 0:49)	20:30 (± 0:28)	0.157
Bedtime on weekends		20:12 (± 0:50)	21:38 (± 1:09)	21:05 (± 1:05)	20:50 (± 0:39)	0.009
Wake time on weekdays		6:36 (± 0:32)	6:42 (± 0:47)	6:36 (± 0:31)	7:23 (± 0:44)	0.006
Wake time on weekends		7:12 (± 0:54)	8:08 (± 1:30)	7:40 (± 1:14)	7:41 (± 0:49)	0.252
Total sleep time on weekdays (hours)		10.7 (± 0.76)	10.0 (± 1.1)	10.2 (± 0.75)	10.8 (± 0.85)	0.057
Total sleep time on weekends (hours)		10.8 (± 1.15)	10.4 (± 0.9)	10.5 (± 1.0)	10.9 (± 0.84)	0.538
**SLEEPING PATTERNS DURING LOCKDOWN**						
Bedtime on weekdays		19:48 (± 0:40)	21:42 (± 1:21)	20:41 (± 3:07)	20:56 (± 0:35)	0.166
Bedtime on weekends		20:12 (± 1:05)	22:20 (± 1:44)	21:44 (± 1:31)	21:08 (± 0:52)	0.010
Wake time on weekdays		7:06 (± 0:53)	8:31 (± 1:57)	7:53 (± 1:15)	7:45 (± 1:03)	0.094
Wake time on weekends		7:18 (± 1:12)	8:54 (± 2:01)	8:12 (± 1:35)	7:50 (± 0:56)	0.069
Total sleep time on weekdays (hours)		11.3 (± 1.2)	10.8 (± 1.3)	10.8 (± 0.92)	10.8 (± 1.1)	0.789
Total sleep time on weekends (hours)		11.1 (± 1.5)	10.6 (± 1.2)	10.5 (± 1.7)	10.7 (± 1.2)	0.832
**SCREEN USE**						
Daily screen time BEFORE the pandemic > 4 h		0	5 (14%)	1 (2%)	0	0.088
Screen use was for education		0	3 (9%)	5 (11%)	5 (42%)	0.017
Daily screen time DURING LOCKDOWN > 4 h		2 (40%)	28 (82%)	35 (75%)	1 (8%)	< 0.001
Screen time increased in lockdown		3 (60%)	29 (85%)	45 (96%)	2 (17%)	< 0.001
Screen use was for education		1 (20%)	22 (65%)	27 (57%)	3 (25%)	0.042

Data are presented as mean (SD) or count (column proportion) unless otherwise specified. Comparisons were made using ANOVA for continuous data and Kruskal–wallis for categorical variables

While screen time increased in children in all school types, the group with the largest proportion who had increased daily screen time in lockdown were those in private school (96%) when compared to 60% of those not in school yet, 85% of those in public school and 17% of those home-schooled, *p* < 0.001.

### School type when the schools re-opened

**Fig. 2:**
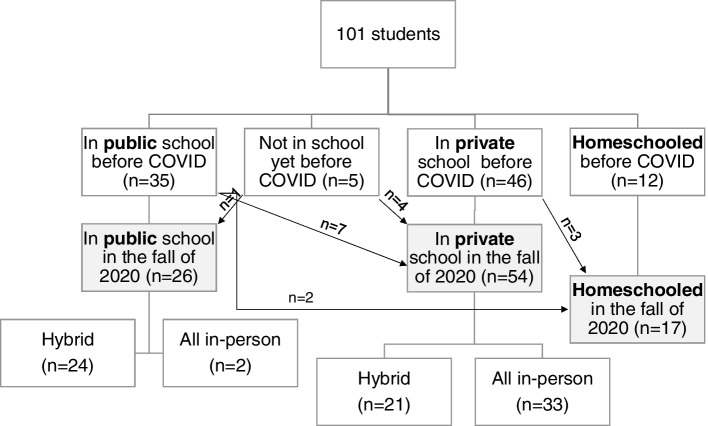
Flow chart of the participants’ school type before the pandemic and when the schools reopened in the fall of 2020. Not all respondents answered the school status at each point in the pandemic so missing answers were omitted from this flow chart

Of the children in private school before the pandemic, only 7% (*n* = 3) changed school type during the fall of 2020; all went to home schooling. Of the children in public school prior to the pandemic, 26% changed school type in the fall with most (7 out of 9) going to private school where the majority (5 of 7) received all in-person schooling (Fig. 2).

Regardless of school type, the majority of students (64%) were in hybrid, virtual or home-schooled when the school year started in fall of 2020. Table 4 shows the comparison between children who were home-schooled, those receiving hybrid or all virtual classes (listed as hybrid) and those receiving all in-person education. The average amount of sleep tended to be higher in home-schoolers when compared to those in hybrid or in-person education (10.9 h versus 10.3 and 10.2, *p* = 0.057) as wake time was later on weekdays (7:42 ± 1:01 vs 7:14 ± 1:09 and 6:41 ± 0:36, *p* = 0.042) respectively.

**Table 4:** Sleep characteristics and screen time in 97 children attending in-person, hybrid or being home schooled during the beginning of the 2020–2021 school year

	Home-schooled (*n* = 17)	Hybrid/virtual (*n* = 45)	All in-person (*n* = 35)	*p*-value
Mean age (years)	8 (± 1.9)	8.8 (± 2.8)	8.4 (± 2.8)	0.595
Average daily hours of sleep	10.9 (± 0.8)	10.3 (± 1.1)	10.2 (± 0.8)	0.057
Average hours of sleep on weekdays	10.9 (± 0.8)	10.2 (± 1.4)	10.1 (± 0.9)	0.241
Average hours of sleep on weekdays	10.9 (± 0.9)	10.7 (± 1.1)	10.5 (± 1.1)	0.794
Bedtime on weekdays	20:49 (± 0:35)	21:06 (± 1:13)	20:36 (± 0:50)	0.680
Wake time on weekdays	7:42 (± 1:01)	7:14 (± 1:09)	6:41 (± 0:36)	0.042
Bedtime on weekends	21:07 (± 0:48)	21:38 (± 1:25)	21:24 (± 1:19)	0.211
Wake time on weekends	7:58 (± 1:12)	8:17 (± 1:45)	7:59 (± 1:36)	0.546
USUALLY or ALWAYS Went to bed the same time every night	10 (59%)	21 (48%)	24 (69%)	0.175
RARELY Slept in another’s bed	16 (94%)	30 (67%)	24 (69%)	0.083
RARELY Had a nightmare	16 (94%)	34 (76%)	22 (63%)	0.052
RARELY Struggled at bedtime	14 (82%)	28 (62%)	26 (74%)	0.241
RARELY Tired or hyperactive during the day	14 (82%)	17 (39%)	11 (31%)	0.002
RARELY Did not get enough sleep	14 (82%)	15 (34%)	16 (46%)	0.003
Sleep quality was GOOD or EXCELLENT	14 (82%)	20 (44%)	22 (63%)	0.020
Screen time > 4 h per day	2 (12%)	34 (76%)	3 (9%)	< 0.001
Screen time > 8 h per day	0	14 (31%)	1 (3%)	< 0.001

Data are presented as mean (± SD) or count (column proportion). Comparisons between continuous data were made using ANOVA and that between categorical variables using Kruskal–wallis testing

A larger proportion of home-schoolers slept well overall. Children in hybrid or in-person schooling had lower rates of getting enough sleep and were more often tired or hyperactive during the day. Per parental report, children in hybrid or virtual learning had the lowest rates of good or excellent sleep quality at 44.4% when compared to that of in-person learners (62.9%) and home-schooled children (82.4%), *p* = 0.020. This equates to home-schoolers having fourfold higher odds of quality sleep when compared to their peers (OR 4.22, 95%CI 1.13–15.84). This association remained significant even after adjusting for daily screen time over 4 h and having good sleep quality before the pandemic (aOR 5.64, 95%CI 1.09–29.08).

As expected by the educational model, more children in hybrid or virtual classes had daily screen time that exceeded 4 h (76%), and even 8 h per day (31%) when compared to home-schoolers (12%, 0%) and to those in all-person learning (9%, 3%), *p* < 0.001. Children with daily screen time less than 4 h had double the odds of good sleep quality (OR 2.66, 95%CI 1.15–6.14).

## Discussion

To date, this is one of the first attempts to describe the granular changes in sleep schedules and sleep quality of school-aged children during different phases of the COVID-19 pandemic in the US. Our data from the beginning of the pandemic are consistent with multiple international reports of later wake and bed times in children [5] and the average amount of increased TST which has been remarkably similar across multiple cohorts [3, 4, 26, 27]. Of note, the importance of 25 min more sleep is clinically questionable, especially relying on self-report, and should be taken in the context that all children’s total sleep time in our study was within the normal recommended hours of 9 to 12 per day for school-aged children.

Importantly, our findings add two previously under-reported aspects of sleep in children during this time. First, our results provide the observation that while total sleep time and sleep habits did improve when schools re-opened in the fall of 2020, they did not return to pre-pandemic levels. No other pediatric sleep study has compared sleep patterns in children from during school closures to when schools re-opening for us to compare our findings. There was a large self-reported survey of over 5,000 adolescents in the United States in the fall of 2020 that found changes in bedtime and wake time when not in structured in-person learning that were consistent with our results [28]. While recall bias could have affected parental report of pre-pandemic sleep (over 9 months prior to survey completion) at the same time as sleep patterns during the ongoing pandemic, even if this is an overstatement of the true effect size, it does suggest that further longitudinal studies are needed to describe the ongoing effects of the pandemic on the sleep health, quality and duration of children past the lockdown period.

Secondly, the increase in total sleep time did not equate to better sleep quality. While there are data in adults that describe the characteristics of pandemic sleep outside of total duration [2, 24, 29], to our knowledge there are few other pediatric studies with a detailed description of the quality of children’s sleep. One was a qualitative study of 37 mothers of preschool children in Italy that reported mother’s perceptions of their child’s appearance upon waking was worse in the first month of lockdown [30]. Our findings support that observation and add more descriptors to this important topic.

One of the possible explanations for decreased sleep quality despite increased sleep time could have been the increase in screen time overall. Previous studies have shown average daily screen time has reportedly increased by 2–4 h in school children during this pandemic [3, 27, 31], and each hour of screen time has a negative linear association with sleep duration in children during lockdown [27]. The connection between screen time and poor sleep quality has been assumed in this pandemic but not well described. Our results show children in virtual learning platforms had the highest rates of excessive screen time while having poorer sleep quality, more inadequate sleep and more daytime tiredness or hyperactivity. These are findings seen in international studies in school-aged children in Italy and Canada [32]. Protecting bedtime from screen exposure was one of the key components of ways to protect children’s sleep health during the pandemic in the health advisories released in 2021 by the American Academy of Sleep Medicine and the National Sleep Foundation [33, 34]. Our data underscore this important message.

There appear to be sub-populations of children more vulnerable to poor sleep from the pandemic that could be considered as targets for future intervention. Consistent with international data, we found older children had larger swings in total sleep time during lockdown that reverted back to less sleep when school restarted in the fall [4, 27, 35]. This mirrors the phenomenon that causes “social jet lag” from early school start times and leads to insufficient sleep in teenagers. The type of educational model also appears to be important. Home-schoolers, who can set their own school schedules, did better in all phases of the pandemic studied. Prior to the pandemic, homeschoolers had later wake time and bedtimes with increased TST and higher quality sleep than public or private school students [36]. Our findings suggest that future educational and interventional efforts to protect the sleep of those most vulnerable to large changes should target the sleep of adolescents and children in public or private schools.

Our data are limited by the small sample size and cross-sectional nature of the study design. While other pandemic studies have used this design because of the challenges of performing research in a public health crisis, it does introduce recall bias in self-reported sleep habits by describing three time points in one survey at one time. Additionally, to limit the burden on the respondents and ensure survey completion was feasible, we opted not to include screens for depression or emotional difficulties in this study. This limited our ability to adjust the associations we describe for any anxiety or stress in the study population.

## Conclusions

When schools closed at the beginning of the COVID-19 pandemic, overall sleep time increased in school children while sleep quality decreased; trends which improved but did not normalize when schools re-opened in the fall of 2020. Older children and those in hybrid learning were most impacted by schedule changes and increased screen time both during lockdown and school restart. Screen time is associated with poor sleep quality. Longitudinal studies are needed to measure the ongoing impact of this pandemic on all dimensions of children’s sleep health.

## Supplementary Information

**Additional file 1.**

## Data Availability

The datasets used and/or analyzed during the current study are available from the corresponding author on reasonable request.

## Abbreviations

COVID-19: Coronavirus disease 2019
TST: Total sleep time
