# Supplementary material for: Parental survey of the sleep patterns and screen time in US school children during the first 6 months of the COVID-19 pandemic
Source: BMC Pediatr. 2023 Feb 8;23:65. doi: 10.1186/s12887-023-03875-9 (PMC9905756; doi:10.1186/s12887-023-03875-9)
Supplement: Supplementary file 1 — Additional file 1. [file 12887_2023_3875_MOESM1_ESM.pdf]

Parental survey on COVID and sleep health in school-aged children

You are invited to participate in a research project being performed by the UBMD Pediatrics Sleep Center to measure how the COVID 19 pandemic has impacted the sleep of school-aged children throughout our community. By entering the survey, you are consenting to participate. No personal information is being collected. All answers are anonymous.

Please complete the survey below to describe how ONE of your children has been sleeping during this difficult time. You can complete multiple surveys if you have multiple school-aged children. We appreciate any time you can provide us.

If you have any additional questions concerning this research or your participation in it, please feel free to contact us or our university research office at any time at 716.323.0370. This research has been reviewed and approved by an Institutional Review Board (“IRB”). An IRB is a committee that provides ethical and regulatory oversight of research that involves human subjects. You may talk to them at (716) 888-4888 or email ub-irb@buffalo.edu.

Thank you!

Drs. Hassinger, Perez and Monegro

UBMD Pediatrics Sleep Center

The survey consists of questions about your child's sleep before COVID (Winter 2019-2020), during the lockdown (March 2020 to June 2020) and NOW, during the start of the 2020 school year (Fall 2020).

If you have more than one child aged 5 to 13, please complete one survey for each child. Try to answer the questions thinking of a typical week during each time period to give us an idea of how COVID has affected your child's sleep patterns.

Have you completed this survey before for another child?	◯ Yes<br>◯ No
In years, how old is the child that you are describing in this survey?	◯ 5 ◯ 6 ◯ 7 ◯ 8<br>◯ 9 ◯ 10 ◯ 11 ◯ 12<br>◯ 13<br>(As of today's date)
What is your child's gender?	◯ Male<br>◯ Female
What is the type of home in which the child lives for the majority of his or her time?	◯ Apartment or condominium<br>◯ Townhouse or split level<br>◯ Semi-detached house<br>◯ Single family home
Which of the following choices best describes the living situation of your child?	◯ Single parent home<br>◯ Single parent with extended relatives at home (aunt, uncle, grandparent)<br>◯ Two parents in the same house<br>◯ Splits time between different homes because parents are not together

What is the total household income of home where the child spends the majority of his or her time?	◯ Less than $25,000 per year<br>◯ Between $25,000 and $49,999 per year<br>◯ Between $50,000 and $99,999 per year<br>◯ Between $100,000 and $149,999 per year<br>◯ Between $150,000 and $249,999 per year<br>◯ $250,000 per year or more<br>◯ Prefer not to say
Are any of the adults in your child's house considered an "essential worker"?	◯ Yes ◯ No

Please answer the following questions about your child's schooling and sleep habits BEFORE the pandemic began, in the winter of 2019-2020.

What type of school did your child attend BEFORE the pandemic?	◯ Not in school yet, too young<br>◯ Public school<br>◯ Charter school<br>◯ Private or Catholic school<br>◯ Home schooled<br>(Choose one that best describes your child's school)
How many hours of screen time did your child have in a typical weekday BEFORE the pandemic?	◯ 0-1 hour per day<br>◯ 1-3 hours per day<br>◯ 4-8 hours per day<br>◯ More than 8 hours per day<br>(This includes computer, tablet, phone and TV)
Was the majority of the screen time (more than half) related to school activities?	◯ Yes ◯ No
BEFORE the pandemic, was your child's screen time different on weekends?	◯ Yes ◯ No
How many hours of screen time did your child have on a typical weekend BEFORE the pandemic?	◯ 0-1 hour per day<br>◯ 1-3 hours per day<br>◯ 4-8 hours per day<br>◯ More than 8 hours per day<br>(This includes computer, tablet, phone and TV)
What time did your child go to bed on a typical weekday/school night BEFORE the pandemic?	________________________<br>(Use the 24 hour clock (Ex: 8:30 pm = 20:30))
BEFORE the pandemic, was your child's bedtime different on weekends?	◯ Yes<br>◯ No
On a typical weekend BEFORE the pandemic, what time did your child go to bed?	________________________<br>(Use the 24 hour clock (Ex: 8:30 pm = 20:30))
BEFORE the pandemic, around what time did your child wake up on a weekday?	________________________<br>(Using the 24 hour clock, so 6am would be 06:00)
Did they wake up at a different time on weekends?	◯ Yes<br>◯ No

BEFORE the pandemic, around what time did your child wake up on the weekends?

____
(Using the 24 hour clock, so 6am would be 06:00)

Choose the frequency for each of the items below related to your child's bedtime patterns BEFORE the pandemic:

	Rarely (0 to 1 time per week)	Sometimes (2 to 4 times per week)	Usually (5 or more times per week)
Went to bed at the same time every night	◯	◯	◯
At any point in the night, would sleep in a parent or sibling's bed	◯	◯	◯
Had a bad dream or nightmare	◯	◯	◯
Struggled at bedtime (cried, refused to stay in bed, etc)	◯	◯	◯
Seemed tired or hyperactive during the day	◯	◯	◯
Did not get enough sleep	◯	◯	◯

How would you describe your child's overall sleep BEFORE the pandemic?

◯ Poor
◯ Fair
◯ Good
◯ Excellent

Please think of a typical week for your child during the beginning of the pandemic in Buffalo when schools were closed (March through the summer of 2020).

What time did your child go to bed on a typical weekday DURING the pandemic lock down?

____
(Use the 24 hour clock (Ex: 8:30 pm = 20:30))

DURING the lockdown, was your child's bedtime different on weekends?

◯ Yes
◯ No

On a typical weekend, what time did your child go to bed?

____
(Use the 24 hour clock (Ex: 8:30 pm = 20:30))

DURING the lockdown, what time did your child wake up on weekdays?

____
(Using the 24 hour clock, so 6am would be 06:00)

Was this different on weekends?

◯ Yes
◯ No

DURING the lockdown, what time did your child wake up on weekends?

____
(Using the 24 hour clock, so 6am would be 06:00)

How many hours of screen time did your child have DURING the pandemic lock-down?

◯ 0-1 hours per day
◯ 1-3 hours per day
◯ 4-8 hours per day
◯ More than 8 hours per day
(This includes computer, tablet, phone and TV)

Was the majority of the screen time (more than half) during this period related to school activities?

◯ Yes ◯ No

Chose the frequency for each of the items below related to your child's bedtime patterns DURING the lockdown phase of the pandemic, from March through the summer of 2020:

	Rarely (0 to 1 time per week)	Sometimes (2 to 4 times per week)	Usually (5 or more times per week)
Went to bed at the same time every night	◯	◯	◯
At any point in the night, would sleep in a parent or sibling's bed	◯	◯	◯
Had a bad dream or nightmare	◯	◯	◯
Struggled at bedtime (cried, refused to stay in bed, etc)	◯	◯	◯
Seemed tired or hyperactive during the day	◯	◯	◯
Did not get enough sleep	◯	◯	◯

How would you describe your child's overall sleep DURING the first 6 months of the pandemic?

◯ Poor
◯ Fair
◯ Good
◯ Excellent

The last set of questions relates to how your child has slept since the school year has started in the fall of 2020.

As all local school districts and private schools have adopted different models for schooling based on space and safety planning, we ask you to describe how often your child attends in-person schooling (meaning physically goes to a school building) or does remote learning (any virtual classroom, synchronous or asynchronous).

Did you change the type of school your child was attending because of the pandemic?

◯ Yes ◯ No

What type of school is your child attending NOW in the fall of 2020?

◯ Public school
◯ Charter school
◯ Private or Catholic school
◯ Home schooled
(Choose one that best describes your child's school)

How many days per week does your child attend in-person classes?	◯ 0 ◯ 1 ◯ 2 ◯ 3 ◯ 4 ◯ 5
How many days per week does your child attend virtual classes?	◯ 0 ◯ 1 ◯ 2 ◯ 3 ◯ 4 ◯ 5
How many hours of screen time does your child get in a typical weekday NOW in the fall of the 2020 school year?	◯ 0-1 hour per day<br>◯ 1-3 hours per day<br>◯ 4-8 hours per day<br>◯ More than 8 hours per day<br>(This includes computer, tablet, phone and TV)
Does your child get different amounts of screen time on weekends now?	◯ Yes ◯ No
How many hours of screen time per day does your child get on weekends NOW in the fall of the 2020 school year?	◯ 0-1 hour per day<br>◯ 1-3 hours per day<br>◯ 4-8 hours per day<br>◯ More than 8 hours per day<br>(This includes computer, tablet, phone and TV)
Is the majority of the screen time (more than half) related to school activities NOW?	◯ Yes ◯ No
What time does your child go to bed on a typical weekday NOW during the school year?	________________<br>(Use the 24 hour clock (Ex: 8:30 pm = 20:30))
Is your child's bedtime different on weekends now?	◯ Yes ◯ No
NOW during the school year, what time does your child go to bed on weekends?	________________<br>(Use the 24 hour clock (Ex: 8:30 pm = 20:30))
What time is your child waking up now on weekdays?	________________<br>(Using the 24 hour clock, so 6am would be 06:00)
Is this different on weekends?	◯ Yes<br>◯ No
What time dose your child wake up on weekends NOW?	________________<br>(Using the 24 hour clock, so 6am would be 06:00)

Choose how often each of the bedtime behaviors below are happening to your child NOW during the fall 2020 school year:

	Rarely (0 to 1 time per week)	Sometimes (2 to 4 times per week)	Usually (5 or more times per week)
Goes to bed at the same time every night	◯	◯	◯

At any point in the night, sleeps in a parent or sibling's bed	○	○	○
Has a bad dream or nightmare	○	○	○
Struggles at bedtime (cries, refuses to stay in bed, etc)	○	○	○
Seems tired or hyperactive during the day	○	○	○
Does not get enough sleep	○	○	○

How would you describe your child's overall sleep NOW?
○ Poor
○ Fair
○ Good
○ Excellent

Has anyone in your child's life had COVID? ○ Yes ○ No

Owens JA, Spirito A, McGuinn M. The Children's Sleep Habits Questionnaire (CSHQ): psychometric properties of a survey instrument for school-aged children. Sleep 2000, Dec 15; 23 (8):1043-51
